# Prognostic value of white matter lesion shrinking in early multiple sclerosis: An intuitive or naïve notion?

**DOI:** 10.1002/brb3.1417

**Published:** 2019-09-26

**Authors:** Viola Pongratz, Paul Schmidt, Matthias Bussas, Sophia Grahl, Christian Gaser, Achim Berthele, Muna‐Miriam Hoshi, Jan Kirschke, Claus Zimmer, Bernhard Hemmer, Mark Mühlau

**Affiliations:** ^1^ Department of Neurology School of Medicine Technical University of Munich Munich Germany; ^2^ School of Medicine TUM Neuroimaging Center Technical University of Munich Munich Germany; ^3^ Department of Psychiatry and Department of Neurology Jena University Hospital Jena Germany; ^4^ Department of Neuroradiology School of Medicine Technical University of Munich Munich Germany; ^5^ Munich Cluster for Systems Neurology (SyNergy) Munich Germany

**Keywords:** demyelinating diseases, magnetic resonance imaging, multiple sclerosis, white matter lesion

## Abstract

**Background and purpose:**

New or enlarging T2‐hyperintense white matter lesions (WML) are associated with clinical disease progression in multiple sclerosis (MS). The prognostic value of WML shrinking is unclear. Assuming that waning of acute inflammation and repair processes would be the main drivers of WML shrinking, we aimed to assess the prognostic value of WML shrinking in early MS.

**Methods:**

We retrospectively analyzed a cohort of 144 early MS patients with three brain MRI scans at baseline and after 1 and 3 years available. All patients were therapy naïve at baseline and 70.5% of them treated with disease modifying drugs at year 1. We determined the volume of WML shrinking between MRI scans, total WML volumes, number of gadolinium‐enhancing and new WML, white matter (WM) and gray matter volumes at each MRI scan. Clinical disability was measured by Expanded Disability Status Scale. We performed the correlation analyses of WML shrinking with other MRI parameters and clinical outcome.

**Results:**

White matter lesions shrinking was highly variable between patients and correlated with the initial number of gadolinium‐enhancing WML and with WM volume decrease. WML shrinking was not associated with clinical outcome.

**Conclusion:**

We found no indication of a prognostic value of WML shrinking in early MS patients. WML shrinking seems to be related to waning of acute inflammation.

## Graphical abstract

White matter lesion (WML) shrinking is highly variable between multiple sclerosis patients and correlates with markers of acute inflammation and with white matter volume decrease. WML shrinking is not associated with clinical outcome.

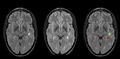

## 1 INTRODUCTION

Number and volume of T2‐hyperintense white matter lesions (WML) are important paraclinical parameters to monitor disease progression and response to immunomodulatory treatment in multiple sclerosis (MS). A high WML load at disease onset is associated with an unfavorable outcome (Tintore et al., 2015). Increase of WML load in a patient under immunomodulatory treatment is associated with treatment failure (Sormani & Bruzzi, 2013). In clinical practice, however, physicians do not only observe newly appearing WML or enlarging WML but also shrinkage or even disappearance of WML. This phenomenon is highly variable from patient to patient. Assuming that the resolution of acute inflammation and repair processes underlie WML shrinking, patients and clinicians may intuitively regard it a good sign. However, the prognostic value of WML shrinking is unclear.

**Figure 1:**
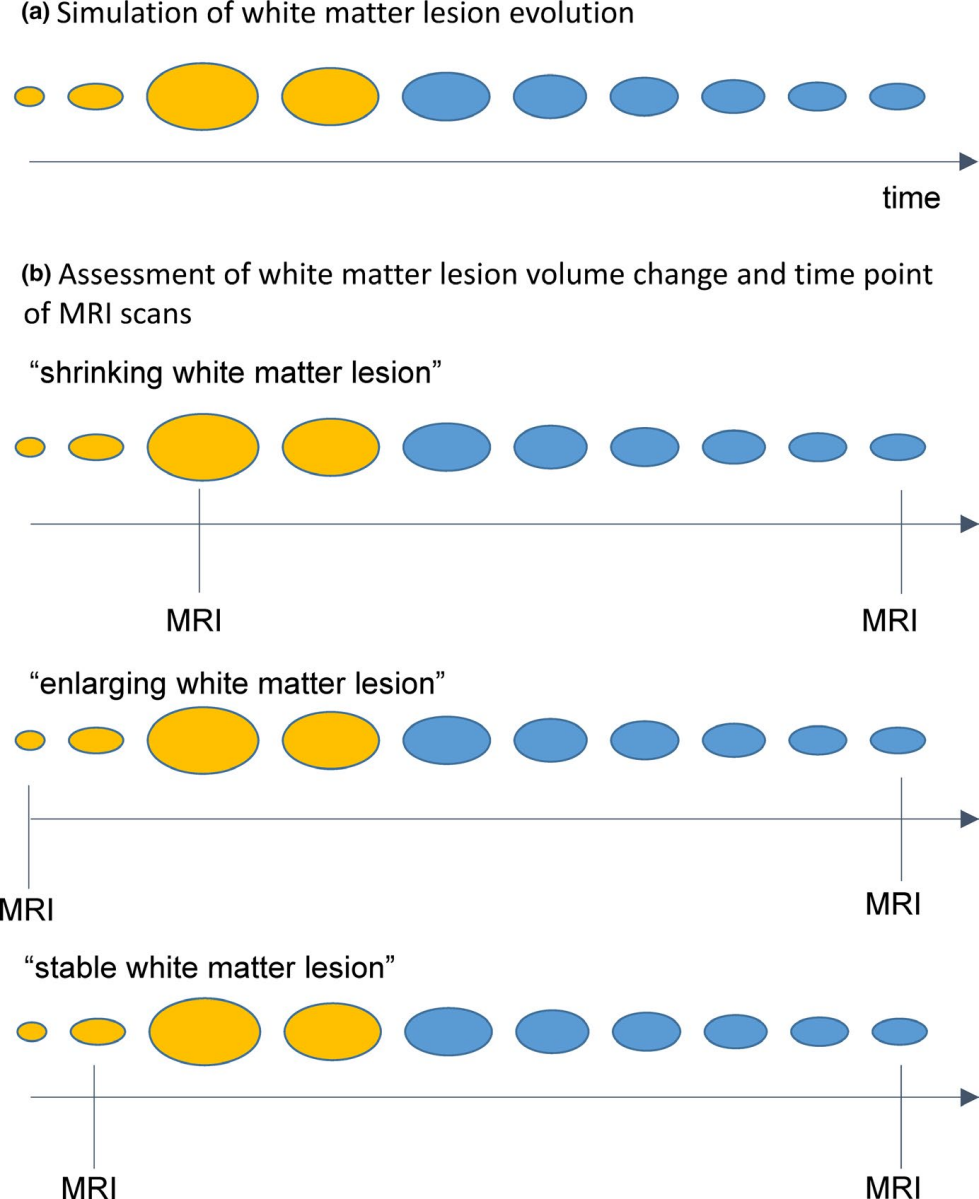
(a) A conceptual scheme of white matter lesion evolution is shown. (b) By three scenarios the dependency of the assessment of white matter lesion volume change on the time point of MRI scans is illustrated. The time points of MRI scans are indicated by a vertical black line. Gadolinium enhancement is illustrated by yellow coloring. Timing of the initial MRI scan may decide whether analysis of the same WML after 1 year demonstrates shrinkage (b, top panel), enlargement (b, middle panel), or stability (b, bottom panel)

We assumed that a newly appearing WML first rapidly increases in size and then slowly decreases (Figure 1a; Meier & Guttmann, 2003; Reich et al., 2015). WML appearance on MRI is accompanied by gadolinium enhancement, which usually lasts for 2–8 weeks (Absinta, Sati, & Reich, 2016; Cotton, Weiner, Jolesz, & Guttmann, 2003; Guttmann et al., 2016; Lai et al., 1996). Determination of WML shrinkage may be erratic in case of a newly appearing WML (Figure 1b). As the first MRI scan may capture different phases of the initial WML growing, timing of the initial scan may decide whether analysis of the same WML after 1 year demonstrates shrinkage (Figure 1b, top panel), enlargement (Figure 1b, middle panel), or stability (Figure 1b, bottom panel).

In our study, we addressed the phenomenon of WML shrinking as observed in daily clinical practice. We investigated a cohort of 144 early MS patients which had received brain MRI scans at baseline (MRI 0), after 1 (MRI 1), and after 3 years (MRI 3). We determined WML shrinking by the longitudinal pipeline of lesion segmentation tool (LST) which measures changes of each individual WML between the consecutive MRI scans. We correlated WML shrinking (MRI 0–1; MRI 1–3) with basic and other MRI parameters to understand its relation to other aspects of MS pathology and to identify potential confounders. Finally, we related WML shrinking to clinical outcome and therapeutic success. To exclude the confounding effect of newly appearing WML as described above, we repeated analyses with clinical parameters in the subgroup of patients without gadolinium‐enhancing WML.

## 2 MATERIALS AND METHODS

### 2.1 Subjects and study design

This study was performed in accordance with the Code of Ethics of the World Medical Association (Declaration of Helsinki) for experiments involving humans and was approved by the local ethics committee. We retrospectively analyzed the data that were collected in an observational study (TUM‐MS) since January 2009 at the Department of Neurology at the Technical University of Munich. We searched for patients that fulfilled the following inclusion criteria: patients were diagnosed with MS according to the 2010 revision of the McDonald criteria (Polman et al., 2011) or clinically isolated syndrome (CIS), which was defined as first demyelinating event suspicious of MS accompanied by at least two WML typical of MS detected by MRI; patients had three consecutive brain MRI scans at baseline (MRI 0) and after about 1 (mean 11.6 months; range 7–16 months) and 3 years (mean 35.78 months; range 30–42 months); patients were not treated with disease modifying drugs (DMD) at baseline. 70.5% of them were treated with DMDs at MRI 1 according to their individual preferences and suggestion by their treating physician. Exclusion criteria were an immunomodulatory treatment prior to the baseline scan and corticosteroid treatment within 30 days prior to any MRI scan. The full dataset was available in 146 MS patients. One patient was withdrawn from analysis due to an artifact in WML segmentation and another due to several tumefactive demyelinating WML, leading to exceptionally high shrinking of WML between consecutive MRI scans. The final analysis was conducted in 144 MS patients. Disability was quantified by Expanded Disability Status Scale (EDSS) by the treating neurologist in the MS outpatient unit. Most EDSS were performed within 1 week of the MRI scan (MRI 1 123/142, MRI 2 137/144, MRI 3 137/144). If no EDSS was available within 1 week and if the patient reported clinical stability, we also accepted EDSS scores performed within 60 days before and after the MRI scan. Demographic and clinical data of the cohort are summarized in Table 1.

**Table 1:** Demographic data and clinical examination

Demographic data			
Sex (male; female)	49; 95		
	**MRI 0**	**MRI 1**	**MRI 3**
Age (years; mean ± *SD*)	35.8 ± 9.9	36.9 ± 9.9	38.8 ± 9.9
Disease duration (years; mean ± *SD*)	1.3 ± 2.9	2.3 ± 2.9	4.3 ± 2.9
Disease course (CIS; RRMS; SPMS)	80; 64; 0	36; 107; 1	17; 125; 2
Disease modifying drugs	None 144	None 42; Baseline therapy 100 (DMF 1; GA 32; IFN 66) Escalation therapy 3 (FTY 1; Nat 2)	None 35; Baseline therapy 95 (DMF 20; GA 25; IFN 48; TFN 1) Escalation therapy 15 (FTY 11; Nat 3; RTX 1)
Clinical examination			
EDSS (*N*; median; range)	137; 1.0; 0–6.0	139; 1.0; 0–6.5	136; 1.0; 0–6.5

Abbreviations: CIS, clinically isolated syndrome; DMF, dimethyl fumarate; EDSS, Expanded Disability Status Scale; FTY, fingolimod; GA, glatiramer acetate; IFN, beta interferon; Nat, natalizumab; RRMS, relapsing‐remitting multiple sclerosis; RTX, rituximab; *SD*, standard deviation; SPMS, secondary progressive multiple sclerosis; TFN, teriflunomide

### 2.2 Magnetic resonance imaging

#### 2.2.1 Scanning protocol

All brain images were acquired on the same 3T scanner (Achieva, Philips, Netherlands). The scanning protocol included 3D GRE T1‐weighted (w) sequence before and after gadolinium injection (orientation, 170 contiguous sagittal 1 mm slices, field of view, 240 × 240 mm; voxel size, 1.0 × 1.0 × 1.0 mm; repetition time (TR), 9 ms; echo time (TE), 4 ms), and 3D FLAIR sequence (orientation, 144 contiguous axial 1.5 mm slices; field of view, 230 × 185 mm; voxel size, 1.0 × 1.0 × 1.5 mm; TR, 10,000 ms; TE, 140 ms; inversion time, 2,750 ms).

#### 2.2.2 T2‐hyperintense white matter lesion segmentation

T2‐hyperintense WML were segmented from FLAIR and T1‐w images by the lesion growth algorithm as implemented in the lesion segmentation tool (LST) toolbox (Schmidt et al., 2012) version 2.0.15 (http://www.applied-statistics.de/lst.html) for SPM12 (http://www.fil.ion.ucl.ac.uk/spm). The same initial threshold (*κ* = 0.3) was used for all images. This value has been proven to be useful in previous studies(Muhlau et al., 2013; Rissanen et al., 2014) and was confirmed by visual inspection.

Lesion segmentation tool's longitudinal pipeline (Schmidt et al., 2019) was used to assess WML decrease and increase between MRI 0–1, and 1–3, based on changes of each individual WML. The longitudinal pipeline comprises the following steps:

#### 2.2.3 White matter lesion filling

White matter lesion were filled with intensities of normal appearing white matter (WM) in the T1‐weighted images to avoid their negative impact on intrasubject registration (Chard, Jackson, Miller, & Wheeler‐Kingshott, 2010; Sdika & Pelletier, 2009).

#### 2.2.4 Intrasubject registration

For every subject, the filled T1‐weighted images were coregistered to a “halfway space” (Smith, De Stefano, Jenkinson, & Matthews, 2001) by longitudinal rigid registration as currently implemented in the SPM toolbox CAT12 (http://dbm.neuro.uni-jena.de/cat/). This algorithm combines rigid‐body registration with initial bias‐field correction. Coregistration matrices were also applied to corresponding FLAIR images after initial coregistration to the corresponding T1‐weighted image (same subject, same time point).

#### 2.2.5 Longitudinal segmentation

**Figure 2:**
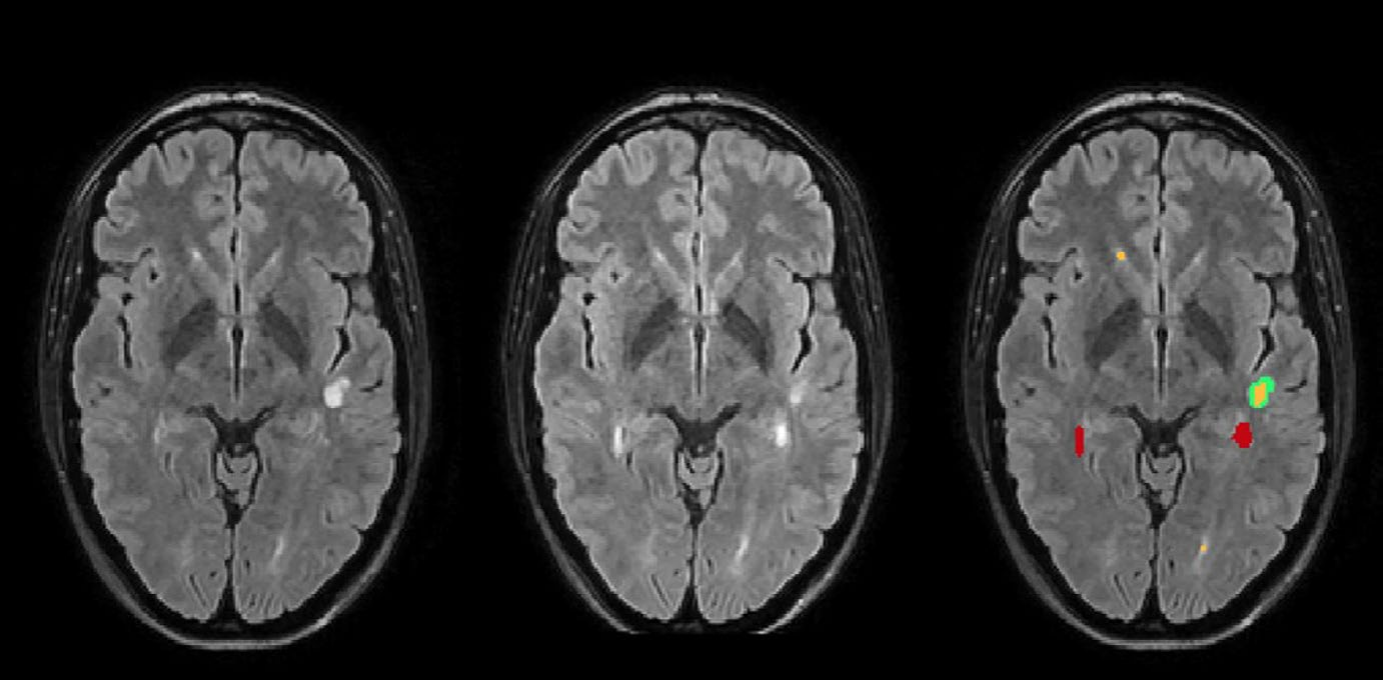
An example of LST's comparison of two time points is shown for one patient. Axial slices of FLAIR images superimposed with white matter lesion maps are shown: left, time point 1; middle, time point 2; right, white matter lesion changes with the following color coding: red, increased; yellow, unchanged; green, disappeared. ml, milliliter

A joint lesion map was created. This is a binary mask including all voxels that were segmented as a WML in at least one time point. For each time point, FLAIR intensities were normalized (scaled) by dividing all voxel values by the mean of segmented gray matter. Then, FLAIR intensities of all consecutive time points were checked for significant changes in the joint lesion map in comparison to changes in normal appearing WM. A lesion change label (WML at both time points, newly appearing WML, disappearing WML) was assigned to each voxel in the joint lesion map for each comparison of consecutive time points. An example of LST's output format is demonstrated in Figure 2. All analyses of WML changes were checked by thorough visual inspection, which confirmed the performance of LST as expected (Schmidt et al., 2012, 2019). Two patients were excluded (one with artifact, one with tumefactive WML).

#### 2.2.6 New and gadolinium‐enhancing T2‐hyperintense white matter lesions

The number of new and gadolinium‐enhancing WML was extracted from the radiology report.

#### 2.2.7 Segmentation of brain volumes

Brain volumes were segmented with the computational anatomy toolbox (CAT12, version 916, http://dbm.neuro.uni-jena.de/cat/) as implemented in SPM12 (SPM12, version 6685). The longitudinal segmentation pipeline was used with the default settings: Lesion‐filled T1‐w images were normalized to Montreal Neurological Institute (MNI) template, segmented into the tissue classes of gray matter (GM) and WM and corrected for signal inhomogeneities (correction of bias field). Segmented images were scaled with the amount of volume changes resulting from normalization (modulation) (Bezzola, Merillat, Gaser, & Jancke, 2011).

### 2.3 Statistical analysis

IBM SPSS Statistics version 25 was used to analyze the data. A natural logarithmic transformation was applied to total WML volumes (MRI 0, MRI 1, MRI 3) to approximate normal distribution. Paired *t* tests were used to investigate differences in total WML volume, WM, and GM volume (MRI 0–1; MRI 1–3). WML shrinking, WML increase, the number of new and gadolinium‐enhancing WML were not normally distributed and therefore compared by related samples Wilcoxon signed‐rank test. Spearman's rank correlation coefficient was used to determine the association between WML shrinking (MRI 0–1; MRI 1–3) with demographic and other imaging parameters. Two linear regression models were performed with WML shrinking (MRI 0–1 and 1–3) as response variable and demographic and imaging parameters as explanatory variables. As the number of gadolinium‐enhancing and new WML was closely related, only the number of gadolinium‐enhancing WML at the initial scan was used in this model. The association of EDSS with WML shrinking was investigated by partial correlation analyses correcting for time interval between MRI 0 and last EDSS and white matter lesion volume at the initial MRI scan. The analyses of WML shrinking (MRI 0–1 and 1–3) with EDSS were repeated in the subgroups of patients without gadolinium‐enhancing WML at all MRI scans (*N* = 87) to exclude the influence of newly appearing WML. To assess the relationship of WML shrinking with therapeutic success, a binary logistic regression analysis was performed with therapy switch due to treatment failure as dependent variable and WML volume shrinking between MRI 0 and 1 as independent variable. Patients treated with escalation therapies at MRI 1 (*N* = 3) were not included in this analysis.

## 3 RESULTS

### 3.1 Brain imaging parameters

Imaging parameters of all time points are summarized in Table 2. Mean WML volume did not change significantly between MRI 0 and 1 and increased significantly between MRI 1 and 3 (*p* < .001). The number of gadolinium‐enhancing WML was significantly higher at MRI 0 compared to MRI 1 (*p* .024) or MRI 3 (*p* < .001). Mean volumes of GM and WM decreased during the study period.

**Table 2:** MRI data

	MRI 0	MRI 1	MRI 3	*p* Value of comparison MRI 0–1	*p* Value of comparison MRI 1–3
Total white matter lesion volume (ml, mean ± *SD*)	3.26 ± 3.99	3.20 ± 4.23	3.61 ± 4.84	.21	<.001
White matter lesion increase (ml; mean ± *SD*)	–	0.36 ± 0.83	0.52 ± 1.19	–	0.056
White matter lesion shrinking (ml; mean ± *SD*)	–	−0.42 ± 0.93	−0.11 ± 0.32	–	<.001
Number of new white matter lesions (mean ± *SD*; range)	–	1.01 ± 1.81; 0–10	2.11 ± 3.69; 0–21	–	<.001
Number of gadolinium‐enhancing white matter lesions (mean ± *SD*; range)	0.73 ± 2.21; 0–16	0.32 ± 1.05; 0–9	0.17 ± 0.74; 0–7	.024	.183
White matter volume (ml; mean ± *SD*)	487.1 ± 56.5	484.2 ± 55.2	481.4 ± 54.1	<.001	<.001
Gray matter volume (ml; mean ± *SD*)	654.1 ± 66.3	648.7 ± 64.9	641.7 ± 62.0	<.001	<.001

Note

Time points were compared by paired *t* test (white matter volume, gray matter volume, total white matter lesion volume) and related samples Wilcoxon signed‐rank test (new white matter lesions, gadolinium‐enhancing white matter lesions, white matter lesion increase, and white matter lesion shrinking).

For MRI 3, changes in number and volume of white matter lesions are given with respect to MRI 1.

Abbreviations: ml, milliliter; MRI, magnetic resonance imaging; *SD*, standard deviation.

### 3.2 Correlation of white matter lesion shrinking with demographic parameters

White matter lesion shrinking between MRI 0 and 1 was associated with DMD treatment at MRI 1 (*r* = −.169, *p* .043), WML shrinking between MRI 1 and 3 was associated with DMD treatment at MRI 3 (*r* −0.230, *p* .006). More effective DMDs were associated with more pronounced shrinking of WML. Age, sex, disease duration, and time interval between MRI scans were not significantly associated with WML shrinking.

### 3.3 Correlation of white matter lesion shrinking with markers of acute inflammation

**Figure 3:**
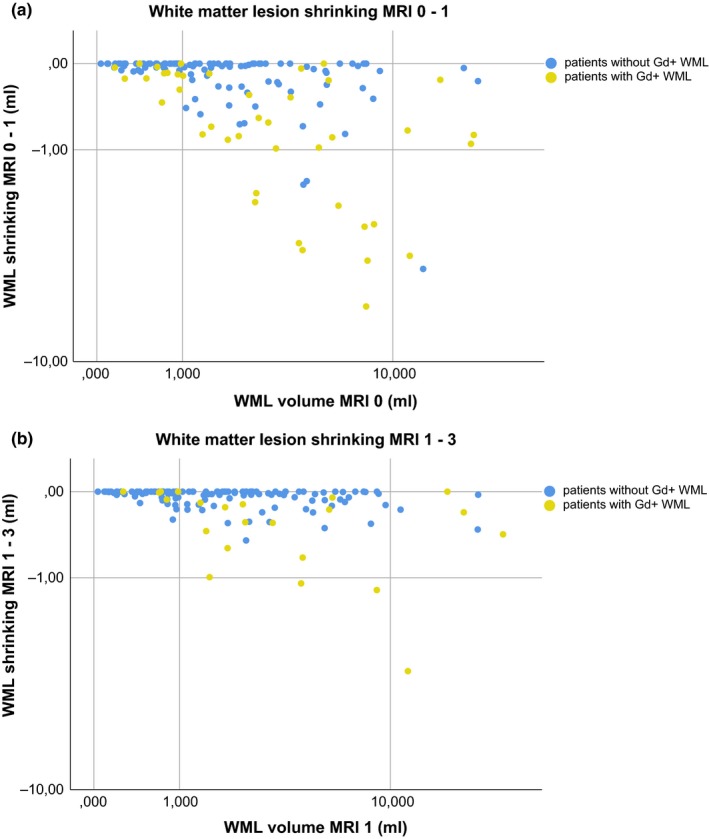
Upper panel: The relation of white matter lesion volume at MRI 0 with white matter lesion shrinking between MRI 0 and 1 is illustrated by a scatter plot. Patients showing gadolinium‐enhancing white matter lesions are illustrated by yellow dots, patients without gadolinium‐enhancing white matter lesions by blue dots. Lower panel: The same graph is shown for the relationship between white matter lesion volume at MRI 1 with decrease in white matter lesion volume between MRI 1 and 3. Scaling of axes, logarithmic; ml, milliliter; WML, white matter lesion

White matter lesion shrinking between MRI 0 and 1 correlated with WML volume at MRI 0 (*r* −.471, *p* < .001; Figure 3a). Highest shrinking of WML volume was found in patients with gadolinium‐enhancing WML at MRI 0 (yellow dots). WML shrinking between MRI 0 and 1 correlated with the number of gadolinium‐enhancing WML at MRI 0 (*r* −.530, *p* < .001).

White matter lesion shrinking between MRI 1 and 3 (Figure 3b) was less pronounced than between MRI 0 and 1 (*p* < .001) and correlated with WML volume at MRI 1 (*r* −.364, *p* < .001). Highest WML shrinking was again found in patients with gadolinium‐enhancing WML at MRI 1. WML shrinking between MRI 1 and 3 correlated with the number of gadolinium‐enhancing WML at MRI 0 (*r* −.286, *p* < .001); it was further associated with the number of new (*r* −.484, *p* < .001) and gadolinium‐enhancing (*r* −0.356, *p* < .001) WML at MRI 1 and the number of new WML at MRI 3 (*r* −0.292, *p* < .001).

### 3.4 Correlation of white matter lesion shrinking with brain volume changes

WML shrinking between MRI 0 and 1 was related to a reduction of WM volume between MRI 0 and 1 (*r* = .290, *p* < .001). WML shrinking between MRI 1 and 3 was related to a reduction of WM volume between MRI 1 and 3 (*r* .447, *p* < .001). No association between WML shrinking and changes of GM volume was found (all *p*‐values > .1).

### 3.5 Multiple linear regression model to explain variance of white matter lesion shrinking

In a linear regression model, WML shrinking between MRI 0 and 1 was explained by age (younger MS patients had more pronounced WML shrinking), WML volume at MRI 0, the number of gadolinium‐enhancing WML at MRI 0 and WM volume change between MRI 0 and 1 (*R* ^2^ 0.362, Table 3). WML shrinking between MRI 1 and 3 was explained by the number of gadolinium‐enhancing WML at MRI 1 and WM volume change between MRI 1 and 3 (*R* ^2^ 0.284, Table 4).

**Table 3:** Regression analysis of white matter lesion shrinking between MRI 0 and 1 with demographic and other MRI parameters

Dependent variable: WML shrinking MRI 0–1	Coefficient	95% Confidence interval		*p*
		Lower bound	Upper bound	
(Constant)	.073	−1.540	1.687	.928
Age	.021	0.006	0.035	**.005**
Sex	−.005	−0.276	0.266	.971
Disease duration	−.009	−0.055	0.037	.687
Time interval MRI 0–1	−.002	−0.006	0.002	.282
Escalation therapy MRI 1	−.090	−0.367	0.187	.522
WML volume MRI 0	−.219	−0.337	−0.102	**.000**
Gd + WML MRI 0	−.139	−0.200	−0.078	**.000**
GM volume change MRI 0–1	.002	−0.008	0.012	.708
WM volume change MRI 0–1	.027	0.001	0.053	**.045**

Abbreviations: DMDs, disease modifying drugs; Gd, Gadolinium; GM, gray matter; MRI, magnetic resonance imaging; n.s., not significant; WM; white matter; WML, white matter lesion

**Table 4:** Regression analysis of white matter lesion shrinking between MRI 1 and 3 with demographic and other MRI parameters

Dependent variable: WML shrinking MRI 1–3	Coefficient	95% Confidence interval		*p*
		Lower bound	Upper bound	
(Constant)	.373	−0.691	1.436	.489
Age	.001	−0.004	0.006	.699
Sex	−.035	−0.137	0.068	.504
Disease duration	.006	−0.011	0.024	.463
Time interval MRI 1–3	−.001	−0.002	0.001	.475
Escalation therapy MRI 3	−.089	−0.180	0.001	.053
WML volume MRI 1	−.032	−0.075	0.011	.145
Gd + WML MRI 1	−.053	−0.100	−0.006	**.026**
GM volume change MRI 1–3	−.001	−0.004	0.002	.516
WM volume change MRI 1–3	.015	0.008	0.022	**.000**

Abbreviations: DMDs, disease modifying drugs; Gd, Gadolinium; GM, gray matter; MRI, magnetic resonance imaging; n.s., not significant; WM; white matter; WML, white matter lesion

### 3.6 Correlation of white matter lesion shrinking with clinical parameters

Expanded Disability Status Scale was low at all three time points (Table 1), did not change significantly during the study period of 3 years (*p* > .3) and correlated with total WML volume exclusively at MRI 2 (*r* 0.216, *p* .01). Therefore, the latest EDSS test performed in our center (status February 2019) was considered to investigate prognostic relevance of WML shrinking. EDSS beyond 3 years after baseline MRI was available in 129 patients (6.87 ± 1.46 years after MRI 0, mean EDSS 1.399 ± 1.490). Last EDSS differed significantly from EDSS at MRI 0 (*p* < .001). Last EDSS was associated with WML volume at MRI 1 (0.189, *p* .033) and MRI 3 (*r* .207, *p* .019) but not with WML shrinking neither between MRI 0 and 1 nor between MRI 1 and 3. Also in the subgroups of patients without gadolinium‐enhancing WML at all MRI scans, WML shrinking was not associated with last EDSS.

**Figure 4:**
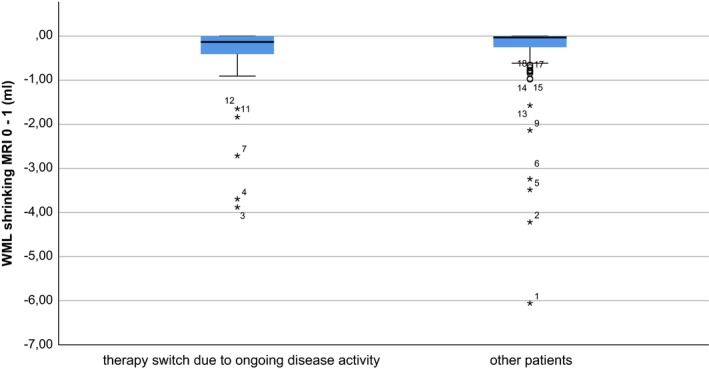
Box plot of WML shrinking between MRI 0 and 1 for patients with therapy switch between MRI 1 and 3 due to ongoing radiological or clinical disease activity (left) and other patients (right; no therapy switch or switch within baseline therapies due to side effects) is shown. Patients are numbered with respect to the extent of white matter lesion shrinking (1 = highest). High WML shrinking between MRI 0 and 1 had no significant effect on therapeutic success (binary logistic regression model, *p* .948). Five patients with pronounced WML shrinking between MRI 0 and 1 were switched between MRI 1 and 3 due to ongoing clinical or radiological disease activity. Patients under escalation therapies at MRI 1 (*N* = 3) are not included

Therapy switch between MRI 1 and 3 was not associated with WML volume at MRI 0 (*p* .126) or WML shrinking between MRI 1 and 3 (*p* .948). The fact that WML shrinking is not associated with a good treatment response is further underlined by the observation that 5 of the 15 patients with the highest WML shrinking between MRI 0 and 1 underwent therapy switch between MRI 1 and 3 due to ongoing clinical or radiological disease activity (Figure 4).

## 4 DISCUSSION

In the study at hand, we aimed to assess the prognostic value of WML shrinking in early MS patients as observed in clinical practice.

Clinicians and patients may intuitively regard WML shrinkage between annual follow‐up scans a good sign. To some degree this idea is naïve, as it is not consistently supported by the current literature: Pathophysiological mechanisms of WML shrinking in early MS patients may be heterogeneous with opposite effects on clinical outcome. On one hand, WML shrinkage could reflect resolution of edema, waning of acute inflammation, and remodeling processes and therefore indicate a good prognosis. On the other hand, it could in part result from resorption of irreversibly destroyed tissue and therefore be an unfavorable sign (Dwyer et al., 2018). It is currently unclear whether the extent of remodeling processes is reflected by changes in T2‐hyperintense WML size. In one histopathological study, even completely remyelinated WML show a hyperintense signal on a T2‐weighted MRI sequence (Barkhof et al., 2003).

We related WML shrinking to markers of acute inflammation and tissue loss. Indeed, we found a robust correlation of WML shrinking with markers of acute inflammation (number of new and gadolinium‐enhancing WML). Since WML shrink most in the first years after their appearance, we believe that early WML shrinking is primarily due to waning of edema and acute inflammation.

In addition, we found a correlation of WML shrinking with reduction of WM volume, which might also be explained by the waning of initial inflammation/edema. This “pseudoatrophy” particularly in the white matter has been described to accompany initiation of several disease modifying drugs (Dwyer et al., 2015; Vidal‐Jordana, Sastre‐Garriga, & Pérez‐Miralles, 2013, 2016; Zivadinov et al., 2008). In addition, the association of WML shrinking with reduction of WM volume could in part reflect degradation of some irreversibly destroyed tissue within the WML and in related regions as had been demonstrated by the novel technique of voxel‐guided morphometry (Fox et al., 2016). In contrast, a long‐term study, investigating WML changes in 22 MS patients over a mean follow‐up period of 16.4 years, found no association of WML shrinking with changes in supratentorial brain volume (Sethi et al., 2016). However, just one WML per patient was considered and related to changes in whole brain but not WM volume. Correspondingly, we found an association of WML shrinking with changes in WM but not GM volumes. This is well conceivable with the notion that GM pathology develops partly independent from WM pathology in MS (Calabrese et al., 2015). We therefore do not assume that the association of WML shrinking with WM volume reduction observed in our study results from global degenerative processes.

We found higher WML shrinking in patients treated with more effective DMDs. This correlation, however, lost significance in the linear regression model after correction for baseline inflammatory activity. We assume that therapy is more likely to be escalated in patients with higher inflammatory disease activity which also shows the higher shrinking of WML.

Although WML shrinking was highest in patients with gadolinium‐enhancing WML, some patients did not show substantial WML shrinking even if gadolinium‐enhancing WML were found at the respective baseline scan (Figure 3). In these cases, first MRI scan might have captured WML in their initial growing phase as explained above (Figure 1b). Alternatively, these “less‐shrinking” gadolinium‐enhancing WML could represent a subgroup of WML in which resolution of inflammation fails and ongoing smouldering inflammation interferes with WML shrinkage (Absinta, Sati, Schindler, et al., 2016).

When relating WML shrinking to clinical parameters, we did not find an indication for a prognostic value of WML shrinking. We assume that in early MS patients, different factors contribute to WML shrinking and exert opposite effects on clinical parameters thereby limiting prognostic value of WML size changes. Possibly, advanced longitudinal studies covering different aspects of WML pathology will disentangle various mechanisms of WML shrinking and leverage their prognostic value.

In summary, our study demonstrates that WML shrinking is highly variable in the first years after diagnosis, which is primarily explained by the extent of initial acute inflammation. Intriguingly, WML shrinking is also associated with a reduction of WM volume which could reflect “pseudoatrophy” and in part degradation of irreversibly destroyed tissue. Accordingly, we found no indication of a prognostic value of WML shrinking. Due to various factors influencing WML size in early MS patients, shrinkage in WML size between follow‐up brain MRI scans should be interpreted with caution.

## CONFLICT OF INTEREST

The authors declare that they have no conflict of interest.

## Data Availability

The data that support the findings of this study are available on request from the corresponding author.
